# Surgical management for caseous calcification of mitral annulus associated with coronary artery disease

**DOI:** 10.1093/icvts/ivaf061

**Published:** 2025-03-12

**Authors:** Shinji Kanemitsu, Shunsuke Sakamoto, Toru Mizumoto

**Affiliations:** Department of Cardiovascular Surgery, Anjo Kosei Hospital, Anjo, Aichi, Japan; Department of Cardiovascular Surgery, Anjo Kosei Hospital, Anjo, Aichi, Japan; Department of Cardiovascular Surgery, Anjo Kosei Hospital, Anjo, Aichi, Japan

**Keywords:** caseous calcification of mitral annulus, mitral annular calcification, calcified amorphous tumour

## Abstract

Mitral annular calcification (MAC) is a common finding, especially among the elderly or patients undergoing haemodialysis. Caseous calcification of the mitral annulus (CCMA) is a rare MAC variant with liquefied material at the calcified annulus. Surgical management of CCMA often involves wide excision and debridement, increasing the risk of perioperative stroke. We report a patient undergoing haemodialysis who developed an enlarged MAC, moderate mitral insufficiency and multivessel coronary artery disease. This case highlights the characteristic imaging features of CCMA. We performed a limited incision for complete drainage, followed by suture obliteration of the cavity and mitral valve repair, in conjunction with coronary artery bypass grafting. This technique was safe, preserved mitral valve function and was not associated with perioperative stroke. We herein report this approach reduces the risk of stroke while maintaining mitral valve function, offering a safer alternative to extensive excision.

## INTRODUCTION

Caseous calcification of the mitral annulus (CCMA) is a rare form of mitral annular calcification (MAC), characterized by a toothpaste-like mass enclosed within the annular region. Although the incidence of CCMA is low, ranging from 0.5 to 1.0% among patients with MAC, it poses significant clinical risks, including systemic embolization and conduction abnormalities [1]. Surgical intervention is often warranted in cases of embolic phenomena, mass enlargement or mitral regurgitation. Reports on surgical management are limited, with many reports requiring valve replacement [2]. Traditional surgical approaches, such as extensive excision and debridement, carry a high risk of complications, including perioperative stroke. We describe a minimally invasive approach using limited incision, drainage, suture obliteration and valve repair. This technique effectively minimizes the embolization risk and preserves valve function, offering a safer alternative.

## CASE DESCRIPTION

**Figure 1:**
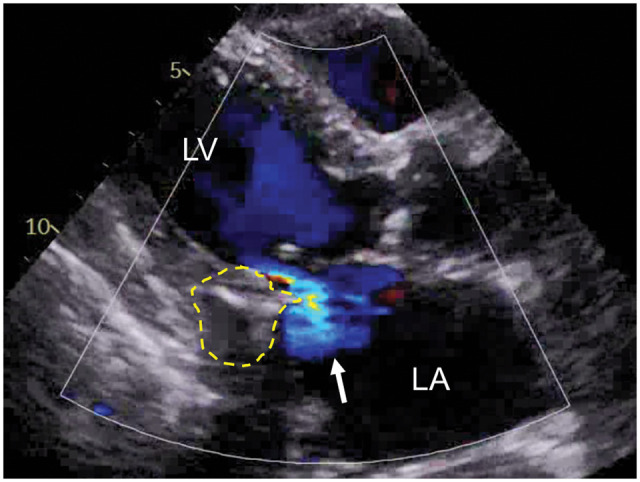
Transthoracic echocardiogram (parasternal long axis view). Preoperative echocardiogram demonstrating an internal echolucent mass with acoustic shadow (dashed line) and mitral valve insufficiency (arrow). LA: left atrium; LV: left ventricle

**Figure 2:**
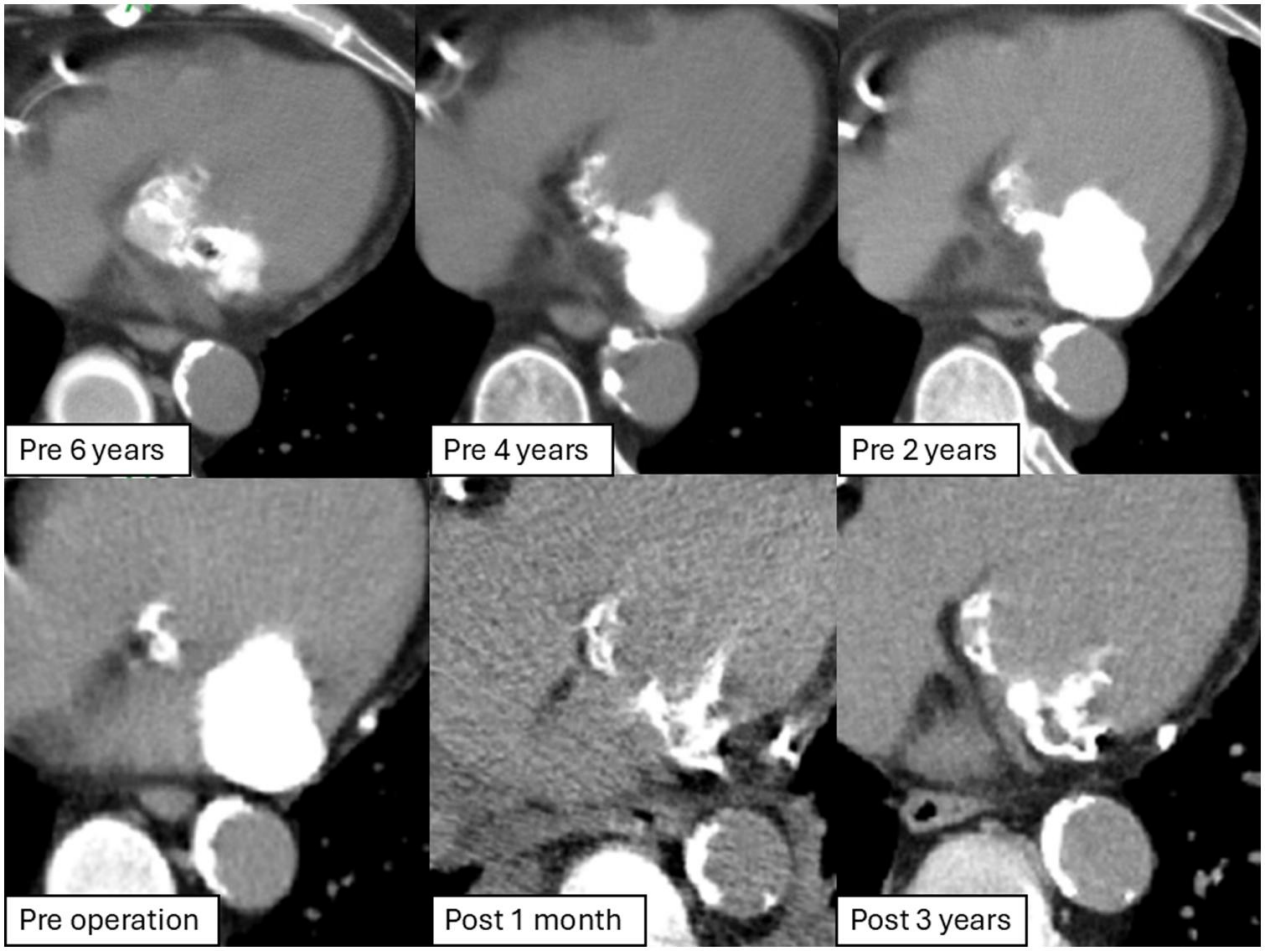
Computed tomography images. CT findings from 6 years preoperatively to 3 years postoperatively. They showed an increase in size of the mass over time, 38×21 mm preoperatively. Postoperatively, there was no evidence of recurrence

A 67-year-old woman with end-stage renal disease on haemodialysis for over 20 years presented with dialysis-related chest pain. Echocardiography revealed the posterior mitral annulus mass, with a smooth surface, central echolucency and associated moderate regurgitation (Fig. 1), consistent with CCMA. Computed tomography (CT) imaging revealed progressive growth of the mass from 10 to 30 mm over 5 years (Fig. 2). The Guerrero CT score as an assessment of MAC was 4 in this case [3]. Coronary artery angiography showed multivessel disease. The patient underwent surgery via median sternotomy. The mass was incised, and toothpaste-like material was aspirated. Complete drainage and decalcification were performed, followed by thorough irrigation. The heavily calcified and friable cavity walls were carefully closed (Video 1). Reducing the filled cavity restored posterior leaflet mobility, allowing successful mitral valve repair. Coronary artery bypass grafting was performed for multivessel disease. Histopathological analysis confirmed the diagnosis of a calcified amorphous tumour. At the 5-year follow-up, the patient remained free from embolic events, with no recurrence, preserved mitral valve function and no exacerbation of mitral regurgitation (Fig. 2).

## DISCUSSION

CCMA, though rare, demands meticulous diagnosis and management due to risks like systemic embolization [1]. Multimodality imaging, combining echocardiography, magnetic resonance imaging and CT, plays a critical role in differentiating CCMA from thrombi, abscesses or tumours [2]. Cardiac magnetic resonance imaging provides superior tissue characterization, enhancing differentiation. The mechanism involved in liquefaction and caseation is unknown. Patients with renal failure are predisposed due to altered calcium-phosphate metabolism, requiring vigilance. The natural history of CCMA are unknown, but we reported that MAC-related calcified amorphous tumour may grow quickly, particularly in patients undergoing haemodialysis [4]. Surgical management is challenging due to dense calcification and risk of ventricular rupture or the circumflex artery injury. We reported good results with complete resection of the entire CCMA and valve replacement using a pericardial patch, effectively preventing embolism [2]. This previous approach serves as a rescue procedure if intraoperative control reveals massive insufficiency, ensuring a safe alternative. Although complete resection with valve replacement has been shown to yield favourable outcomes in patients with severe leaflet degeneration, our case underscores the efficacy of a conservative approach when the CCMA is localized with intact leaflets and subvalvular apparatus. Prioritizing cavity drainage, suture obliteration and valve repair minimizes manipulation of friable CCMA cavity walls, reducing embolic risk and preserving valve function. A minimally invasive approach (via right thoracotomy) may be feasible for isolated CCMA cases without coronary artery disease. However, this technique may not be suitable for patients with annular perforation, extensive calcification invading subvalvular apparatus or friable cavity walls prone to rupture. Innovative techniques, including concentric spiral sutures and a combination of everting and non-everting mattress sutures, have further advanced the surgical management of CCMA. This case highlights the importance of tailoring surgical interventions to the anatomical and pathological characteristics of CCMA, emphasizing minimally invasive strategies to optimize patient outcomes [5]. In conclusion, extensive resection of the CCMA can result in perioperative embolic stroke. Our approach, minimal incision, drainage and suture closure of the cavity, underscores the importance of individualized surgical strategies tailored to the lesion’s characteristics. By prioritizing valve preservation and reducing the risk of perioperative complications, this technique provides a reproducible and safer alternative for managing CCMA.

## Data Availability

The data underlying this article will be shared on reasonable request to the corresponding author.

## References

[1] Chevalier B, Reant P, Laffite S, Barandon L. Spontaneous fistulization of a caseous calcification of the mitral annulus: an exceptional cause of stroke. Eur J Cardiothorac Surg 2011;39:e184–e5. 21376613 10.1016/j.ejcts.2011.01.038

[2] Kanemitsu S, Bessho S, Sakamoto S, Yamamoto N, Ito H, Shimpo H. Calcified amorphous tumor with caseous calcification of mitral annulus in hemodialysis patients. Gen Thorac Cardiovasc Surg 2020;68:1513–6. 32314150 10.1007/s11748-020-01363-w

[3] Guerrero M, Wang DD, Pursnani A et al A cardiac computed tomography-based score to categorize mitral annular calcification severity and predict valve embolization. JACC Cardiovasc Imaging 2020;13:1945–57. 32417332 10.1016/j.jcmg.2020.03.013

[4] Takeuchi T, Dohi K, Sato Y et al Calcified amorphous tumor of the heart in a hemodialysis patient. Echocardiography 2016;33:1926–8. 27516080 10.1111/echo.13335

[5] Chauvette V, Laflamme É, Lafrenière-Bessi V, et al Caseous calcification of the mitral annulus: a role for surgery. Ann Thorac Surg. 2020;109:e441–e4. 31606520 10.1016/j.athoracsur.2019.08.110

